# Neighborhood socioeconomic factors and characteristics correlated with avoidable emergency department visits: A spatial analysis of a Canadian academic hospital

**DOI:** 10.1371/journal.pone.0311575

**Published:** 2024-10-28

**Authors:** Ryan P. Strum, Brent McLeod, Andrew P. Costa, Shawn Mondoux

**Affiliations:** 1 Research Institute of St. Joe’s Hamilton, St. Joseph’s Healthcare Hamilton, Hamilton, Ontario, Canada; 2 Department of Health Research Methods, Evidence and Impact, McMaster University, Hamilton, Ontario, Canada; 3 Hamilton Paramedic Services, Hamilton, Ontario, Canada; 4 Department of Medicine, Division of Emergency Medicine, McMaster University, Hamilton, Ontario, Canada; 5 Department of Emergency Medicine, St. Joseph’s Healthcare Hamilton, Hamilton, Ontario, Canada; Emory University School of Medicine, UNITED STATES OF AMERICA

## Abstract

**Introduction:**

The influence of neighborhood characteristics and socioeconomic status (SES) factors on avoidable emergency department (ED) utilization is not well understood in a universal healthcare system. We examined correlations between these factors and avoidable ED visits at a Canadian academic hospital.

**Materials and methods:**

We conducted a retrospective cohort study using administrative ED data from a hospital in Hamilton, Canada from April 1, 2018 to August 31, 2023, and neighborhood data from the Statistics Canada Census of Population 2021. Avoidable visits were classified using the Emergency Department Avoidability Classification (EDAC), and mapped to neighborhoods using Canadian postal codes. SES was defined primarily based on education attained, household income, employment and housing security. The top 20 postal codes with the highest avoidable ED visits were categorized into quartiles and analyzed for trends using chi-squared tests of spatial association and Spearman rank correlations.

**Results:**

A consistent ordinal trend across quartiles was observed throughout the study period, with quartile 1 representing the lowest avoidable ED visits and quartile 4 the highest. The quartiles were unevenly distributed spatially, though there was a significant association between close proximity to the ED and avoidable visits (X^2^ = 7.07, p <0.05). The quartile with the highest avoidable ED visits (quartile 4) had the greatest proportion of one-person households (35.5%) and one-parent families (37.8%), and showed statistically significant positive correlations with male sex, living alone and having an indigenous identity. Quartile 4 had the highest rates of individuals not completing high school (18.6%, p < 0.05), unemployment (13.7%), households spending greater than 30% of their income on shelter (26.5%), and households earning less than $30,000 annually (16.6%, compared to 8.7% in quartile 1 with the lowest avoidable ED visits).

**Discussion:**

In a universal healthcare setting, lower SES neighborhoods were correlated with higher rates of avoidable ED visits. Targeted interventions that address social determinants of health disparities in neighborhoods with lower SES could reduce the burden of avoidable ED visits, and promote more equitable healthcare utilization.

## Introduction

The relationship between socioeconomic status (SES) and healthcare utilization has long been a focus of health policy to better understand resource use discrepancies within healthcare systems [1, 2]. Lower SES is a key factor influencing quality of life, and is frequently associated with a lack of resources (i.e., childcare, employment, transportation), which can hinder timely and appropriate management of physical and psychological health [3–5]. These social conditions and socioeconomic circumstances have been linked to a higher likelihood of individuals accessing the emergency department (ED) for care, particularly for low acuity conditions [6, 7].

While the association between lower SES and incidence of ED use has been described broadly, this relationship has not been examined in the context of avoidable ED visits within a universal healthcare system, where financial barriers or lack of health insurance coverage are typically not factors. Avoidable visits are ED visits that could have been managed by primary care providers in the community [8]. These visits burden emergency services and can be an indicator of inefficiencies in the healthcare system [8–10]. Seeking ED care for conditions that would not lead to an increased risk of adverse outcomes if delayed for several hours can indicate issues with geospatial primary care accessibility and utilization [11].

There remains a gap in our understanding of how economic and social disadvantages specifically contribute to avoidable ED visits in a universal single-payer healthcare system. Understanding the relationship between different SES neighborhoods and avoidable ED visits could increase our understanding of factors indirectly driving these ED visits, and explain their increasing frequency. If specific SES characteristics in neighborhoods are linked to higher incidences of avoidable ED visits, these findings could inform targeted interventions and policies aimed at reducing a root cause of avoidable ED visits, thereby improving healthcare equity in an overstrained emergency healthcare system.

Our objective was to identify neighborhood and socioeconomic characteristics associated with a high incidence of avoidable ED visits.

## Materials and methods

### Study design

We conducted a secondary retrospective cohort analysis of administrative ED records from an academic hospital in Hamilton, Ontario, Canada, and neighborhood data from the Statistics Canada Census of Population 2021. We adhered to the Strengthening the Reporting of Observational Studies in Epidemiology (STROBE) statement for the reporting of results, shown in S1 Table [12].

### Avoidable emergency department visits

All patients triaged in the ED of the academic hospital between April 1, 2018, and June 30, 2023 were eligible for inclusion. The Emergency Department Avoidability Classification (EDAC) was utilized to categorize ED visits as either avoidable, potentially avoidable or not avoidable [8]. Avoidable ED visits are defined as visits that could have been managed in non-ED care, potentially avoidable visits as visits that could have likely been managed in non-ED care, and non-avoidable visits as visits requiring ED care [8]. For an ED visit to be classified as avoidable or potentially avoidable, patients must have been between 18 and 70 years of age, triaged with a non-emergent acuity (less urgent or non-urgent for avoidable, urgent for potentially avoidable), discharged from the facility, had no specialist physician consultation, and had a main intervention that was primary care-like [8]. ED visits missing necessary variables for categorization were excluded.

### Geospatial quartiles

Neighborhood boundaries were defined using the forward sortation area (FSA), the first three digits of the Canadian postal code. The top 20 neighborhoods with the highest totals of avoidable and potentially avoidable ED visits over the study period were identified. These neighborhoods were grouped into four uniform ordinal quartiles based on absolute counts of avoidable ED visits: quartile 1 (Q1, neighborhoods with the lowest avoidable ED visits), quartile 2 (Q2), quartile 3 (Q3) and quartile 4 (Q4, neighborhoods with the highest avoidable ED visits). Quartile grouping helped mitigate the impact of small sample sizes, facilitated rank-based correlations by ensuring even distribution across groups, and allowed for the use of non-parametric tests, which do not require assumptions about data distribution.

### Variables

We characterized each quartiles avoidable ED visit patient cohort based on age, sex, medical acuity at ED triage, arrival mode and diagnostic category. Acuity was determined using the Canadian Triage and Acuity Scale (CTAS), an ordinal scale from one to five, where one represents the most severe condition (resuscitation) and five the least (non-urgent) [13]. Diagnostic categories was assigned using the International Classification of Diseases, 10^th^ revision [14]. We included neighborhood characteristics totals of sex, age categories, house type, house ownership, persons per home, indigenous identity, and citizenship. The distance from each neighborhood to the academic hospital was calculated as the distance (in kilometres) from the neighborhood geometric centroid (centre point) within each FSA to the ED.

SES is a measure of relative economic resources and social status that can directly and indirectly influence health status and disease development [6]. SES is a multidimensional construct comprised of education, income, employment, food security and housing security. In this study four SES variables were included and categorized based on data availability in the Census of Population 2021: education, income, employment and housing security.

### Data source

To categorize ED visits, data were extracted from the academic hospitals central data repository, an ED incidence database that collects patient care reports from all ED visits. The Health Information Management Department extracted the data on March 13, 2024, and de-identified all data prior to our analysis. Neighborhood and SES variables were extracted on April 18, 2024 from the 2021 Census of Population by Statistics Canada, a national survey with greater than a 98% response rate from all Canadians [15]. The census is conducted every five years by Statistics Canada, a national government agency that produces valid population, resource, economic, society and cultural data. Completing the census is a legal requirement for all Canadian citizens, permanent residents, refugee claimants, and individuals with school or work permits. No investigator had access to any information that could identify individual patients or residents at any time.

### Statistical analysis

We analyzed trends in avoidable ED visits across the four quartiles both overall, and for the first (2018) and last (2023) study years. Descriptive statistics for ED visits in each quartile were presented using frequencies and proportions. To account for population growth within each quartile, we calculated incident rate ratios (IRRs) with 95% confidence intervals (CIs). Quartiles were also visualized geospatially using a map. The association between neighborhood quartile and distance from the ED was assessed using a chi-squared test of spatial association, which determines whether there is a statistically significant association (p < 0.05) between a categorical variable (avoidable ED visits) and spatial units (neighborhoods). Descriptive statistics of each quartile’s neighborhood characteristics were reported using frequencies and proportions, and SES characteristics using proportions. Spearman rank correlations were used to measure the strength and direction of the relationship between quartiles and each level of neighborhood and SES characteristics. Correlations were evaluated based on a range from 1 to -1, with 1 indicating a perfect positive association, -1 a negative positive association, and 0 meaning no association between the ranks, with 95% CIs. Missingness data were reported by variable. Data were managed and analyzed in R statistical software (v4.3.6).

### Ethics

Our study was approved by the Hamilton Integrated Research Ethics Board (HiREB), review reference number 2023–16838. All data utilized in this study were secondary and de-identified before being made accessible to any investigator. For these reasons, the ethics committee waived our need to obtain consent to conduct this research.

## Results

Our study included 201,742 ED visits from the academic hospital, of which 58,530 (29.0%) were classified as avoidable. Of these visits, 48,983 (83.7%) were from the 20 most frequently named neighborhoods. These top 20 neighborhoods were divided into four uniform quartiles based on their rates of avoidable ED visit incidence. Table 1 shows each quartile’s avoidable ED visits. An ordinal trend is observed overall, and in both years of 2018 and 2023, with Q1 contributing the least avoidable ED visits in 2018 (1,044) and 2023 (1,284) while Q4 contributed the most in 2018 (3,624) and 2023 (3,708). Adjusting for neighborhood population size, the incidence of avoidable ED visits continued to follow the ordinal trend. However, Q1, Q2 and Q3 observed similar growth in IRRs from 2018 to 2023 (1.20, 1.11–1.130; 1.20, 1.12–1.29; 1.19 1.12–1.27, respectively), while Q4 reduced (0.97, 0.93–1.01). Females were the majority of avoidable ED attendance, though this was less pronounced in Q3 and Q4. Demographics related to age, arrival mode and diagnostic categories were relatively consistent across the quartiles.

**Table 1:** Quartiles of avoidable emergency department visits, defined as visits manageable in non-ED settings.

	Quartiles of Avoidable ED Visits			
	Q1	Q2	Q3	Q4
**Avoidable ED Visits**				
Total	6,822	8,774	12,047	21,340
2018	1,044	1,308	1,992	3,624
2023	1,284	1,692	2,220	3,708
**Avoidable Visits per 100,000 Residents**				
2018	934	1,061	1,109	2,736
2023	1,122	1,272	1,323	2,653
**Incident Rate Ratio** (95% CI)	1.20 (1.11–1.30)	1.20 (1.12–1.29)	1.19 (1.12–1.27)	0.97 (0.93–1.01)
**Age**, years				
0–17	0 (0.0)	0 (0.0)	0 (0.0)	0 (0.0)
18–39	2,953 (43.3)	4,341 (49.5)	5,598 (46.5)	10,427 (48.9)
40–64	3,185 (46.7)	3,704 (42.2)	5,363 (44.5)	9,140 (42.8)
65 or over	684 (10.0)	729 (8.3)	1,086 (9.0)	1,773 (8.3)
**Sex**				
Male	3,021 (44.3)	3,920 (44.7)	5,743 (47.7)	10,452 (49.0)
Female	3,800 (55.7)	4,850 (55.3)	6,298 (52.3)	10,878 (51.0)
Other/Unknown	1 (0.0)	4 (0.0)	6 (0.0)	10 (0.0)
**Arrival Mode**				
Walk-In	5,675 (83.2)	7,112 (81.1)	9,196 (76.3)	17,275 (81.0)
Ambulance	1,147 (16.8)	1,662 (18.9)	2,851 (23.7)	4,065 (19.0)
**Acuity**, CTAS				
Urgent	5,643 (82.7)	7,233 (82.4)	9,893 (82.1)	16,881 (79.1)
Less Urgent	881 (12.9)	1,168 (13.3)	1,625 (13.5)	3,204 (15.0)
Non-Urgent	298 (4.4)	373 (4.3)	529 (4.4)	1,255 (5.9)
**Diagnostic Category** ^a^				
Mental Health & Behavioural Disorders	740 (10.8)	1,062 (12.1)	1,729 (14.4)	2,516 (11.8)
Diseases of the Respiratory System	327 (4.8)	467 (5.3)	798 (6.6)	1,437 (6.7)
Diseases of the Digestive System	461 (6.8)	512 (5.8)	725 (6.0)	1,018 (4.8)
Diseases of the Musculoskeletal System	490 (7.2)	605 (6.9)	804 (6.7)	1,668 (7.8)
Diseases of the Genitourinary System	507 (7.4)	650 (7.4)	800 (6.6)	1,151 (5.4)
Symptoms, Signs and Abnormal	1,824 (26.7)	2,390 (27.2)	3,050 (25.3)	4,957 (23.2)
Clinical Findings not Elsewhere				
Classified				
Injury, and Consequences of External	1,654 (24.2)	1,961 (22.4)	2,544 (21.1)	5,944 (27.9)
Causes				
Other	819 (12.0)	1,127 (12.8)	1,597 (13.3)	2,649 (12.4)

**Notes**: ED = emergency department, Q1 = quartile 1 (neighborhoods with the lowest avoidable ED visits), Q2 = quartile 2, Q3 = quartile 3, Q4 = quartile 4 (neighborhoods with the highest avoidable ED visits), CI = confidence interval, CTAS = Canadian Triage and Acuity Scale.

^a^ Categorized by the Canadian Emergency Department Information System (CEDIS) using the International Classification of Diseases 10^th^ revision (ICD-10).

**Fig 1:**
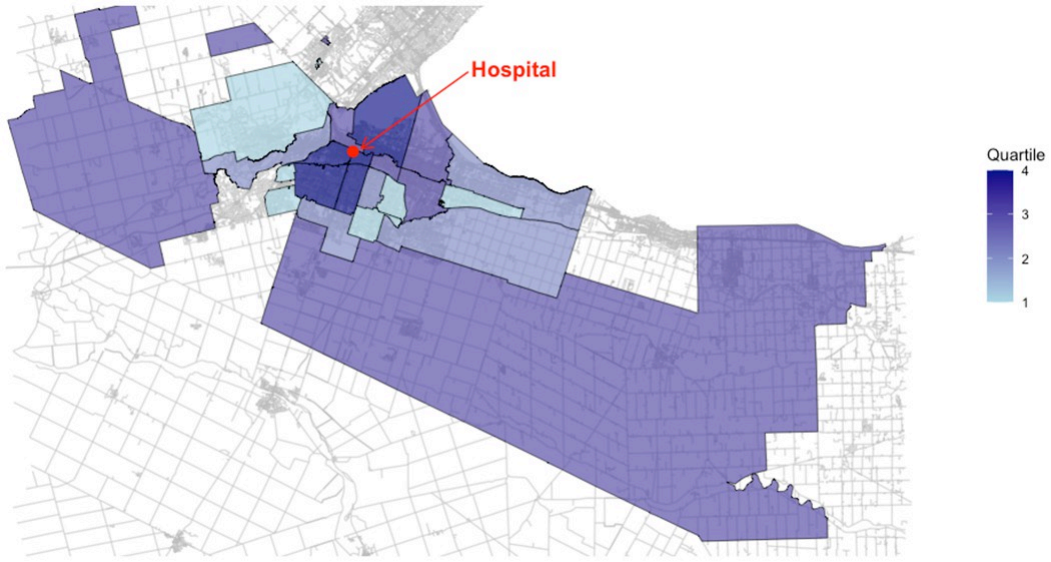
Geospatial map of the four quartiles representing residents with the highest to lowest avoidable ED visits in Hamilton, Ontario, Canada.

Fig 1 presents a map of neighborhoods within each quartile, showing their geospatial distribution and proximity to the academic hospital. Neighborhoods with the most avoidable visits (Q4) are situated in close proximity to the academic hospital, while the remaining quartiles are more dispersed. A chi-squared test for spatial associations determined there was a significant association between the quartiles of avoidable ED visits and distance from the ED (X^2^ = 7.07, p-value <0.05).

Table 2 shows neighborhood statistics of each quartile. Q3 was the most populated with 167,807 residents, while Q1 had the fewest with 114,435. All quartiles exhibited a relatively even distribution across age groups and sex. Quartile 4, which had the highest rate of avoidable ED visits, also had the highest proportion of one-person households (35.3%) and the lowest proportions of households with four (11.1%) or five or more persons (7.9%). Q4 had the lowest percentage of Canadian citizens (86.7%), the highest percentage of one-parent families with children (37.8%), the lowest proportion of single-detached houses (43.9%), the highest proportion of apartments above five stories (26.0%), and the fewest multigenerational households (2.9%). Statistically significant positive correlations were observed from Q1 to Q4 in characteristics of aged 30 to 49 years, male sex, living alone, having an indigenous identity, and certain housing types.

**Table 2:** Neighborhood characteristics and correlations of quartiles derived from avoidable emergency department visit incidence.

	Quartiles of Neighborhoods by Avoidable ED Visits, n (%)				Spearman Rank Correlation	
	Q1	Q2	Q3	Q4	Coefficient (95% CI)	p-value
**Population**	114,435	133,055	167,807	139,750	-	-
**Age**, years						
0–14	17,425 (15.2)	21,615 (16.2)	29,245 (17.4)	20,495 (14.7)	-0.03 (-0.04 –-0.03)	0.96
15–29	20,570 (18.0)	27,110 (20.4)	28,745 (17.1)	29,470 (21.1)	0.41 (0.40–0.41)	0.52
30–49	26,495 (23.2)	33,970 (25.5)	45,830 (27.3)	38,925 (27.9)	0.97 (0.97–0.97)	< 0.05
50–64	25,010 (21.9)	26,230 (19.7)	34,170 (20.4)	27,020 (19.3)	-0.80 (-0.80 –-0.80)	0.15
65–84	20,945 (18.3)	20,580 (15.5)	26,255 (15.6)	20,345 (14.6)	-0.89 (-0.89 –-0.89)	0.08
≥85	3,995 (3.5)	3,525 (2.6)	3,560 (2.1)	3,530 (2.5)	-0.76 (-0.77 –-0.76)	0.18
**Sex**						
Male	55,310 (48.3)	64,925 (48.8)	82,995 (49.5)	70,175 (50.2)	1.00 (1.00–1.00)	< 0.05
Female	59,230 (51.8)	68,130 (51.2)	84,815 (50.5)	69,570 (49.8)	-0.99 (-0.99 –-1.00)	< 0.05
**Persons in Houses**						
1 person	10,235 (23.8)	12,425 (25.1)	18,155 (27.4)	20,810 (35.2)	0.92 (0.92–0.92)	< 0.05
2 persons	14,180 (32.9)	15,105 (30.5)	22,210 (33.5)	18,800 (31.8)	-0.03 (-0.04 –-0.02)	0.96
3 persons	7,320 (17.0)	8,275 (16.7)	10,250 (15.5)	8,345 (14.1)	-0.97 (-0.97 –-0.97)	< 0.05
4 persons	7,010 (16.3)	8,120 (16.4)	9,285 (14.0)	6,570 (11.1)	-0.93 (-0.93 –-0.93)	< 0.05
5 or more persons	4,325 (10.0)	5,570 (11.3)	6,325 (9.6)	4,660 (7.9)	-0.74 (-0.74 –-0.73)	0.20
**Indigenous Identity**	1,525 (1.3)	2,225 (1.7)	4,860 (2.9)	4,115 (2.9)	0.94 (0.94–0.94)	< 0.05
**Citizenship**						
Canadian	105,505 (92.2)	119,050 (89.5)	157,295 (93.7)	121,105 (86.7)	-0.51 (-0.52 –-0.51)	0.41
Not Canadian	7,075 (6.2)	12,010 (9.0)	9,145 (5.4)	15,475 (11.1)	0.55 (0.54–0.55)	0.38
**Family type with Children**						
Married	13,390 (64.6)	15,475 (64.8)	17,215 (59.9)	11,570 (52.2)	-0.92 (-0.92 –-0.92)	0.05
Common-law	1,495 (7.2)	1,665 (7.0)	2,885 (10.0)	2,210 (10.0)	0.88 (0.87–0.88)	0.09
One-parent	5,845 (28.2)	6,730 (28.2)	8,640 (30.1)	8,370 (37.8)	0.87 (0.86–0.87)	0.09
**Number of Houses**	43,065	49,475	66,230	59,210	-	-
**House Type**						
Single-detached	27,320 (63.4)	27,805 (56.2)	40,565 (61.2)	26,020 (43.9)	-0.79 (-0.79 –-0.79)	0.15
Semi-detached	865 (2.0)	1340 (2.7)	2,155 (3.3)	2,560 (4.3)	0.99 (0.99–0.99)	< 0.05
Row house	6,720 (15.6)	8,340 (16.9)	7,115 (10.7)	4,190 (7.1)	-0.90 (-0.91 –-0.90)	0.07
Apartment or flat in a duplex	700 (1.6)	1,195 (2.4)	2,390 (3.6)	3,095 (5.2)	0.99 (0.99–0.99)	< 0.05
Apartment <5 storeys	2,745 (6.4)	2,730 (5.5)	5,990 (9.0)	7,775 (13.1)	0.89 (0.89–0.9)	0.07
Apartment ≥5 storeys	4,655 (10.8)	8,020 (16.2)	7,760 (11.7)	15,395 (26.0)	0.76 (0.76–0.77)	< 0.05
Other	60 (0.1)	45 (0.1)	255 (0.4)	175 (0.3)	0.77 (0.74–0.81)	0.17
**Multigenerational Households**	1,730 (4.0)	2,225 (4.5)	2,260 (3.4)	1,730 (2.9)	-0.81 (-0.82 –-0.81)	0.14

**Note**: ED = emergency department, Q1 = quartile 1 (neighborhoods with the lowest avoidable ED visits), Q2 = quartile 2, Q3 = quartile 3, Q4 = quartile 4 (neighborhoods with the highest avoidable ED visits), CI = confidence interval.

Table 3 shows SES characteristic proportions across neighborhood quartiles. Q4 had the highest proportion of residents who did not finish high school (18.6%). Compared to Q1, Q4 had double the proportion of households earning less than $30,000 annually (16.6% vs. 8.7%), and half the proportion of households earning more than $200,000 (5.8% vs. 12.8%). Q4 had the highest unemployment proportion (13.7%) compared to Q1 (12.2%), Q2 (12.3%) and Q3 (11.6%). Additionally, Q4 had the highest proportion of households spending greater than 30% of their income on housing (26.5%). Overall, Q4 exhibited lower education attainment, household income, employment rates, and housing security compared to Q1.

**Table 3:** Proportions and rank correlations between neighborhood quartiles of avoidable ED visits and socioeconomic status characteristics.

Socioeconomic Status Characteristic	Quartiles of Neighborhoods by Avoidable ED Visits (%)				Q1-Q4 Difference	Spearman Rank Correlation	
	Q1	Q2	Q3	Q4		Coefficient (95% CI)	p-value
**Education**							
Not finished high school	16.2	17.2	18.2	18.6	2.4	0.98 (0.98–0.98)	< 0.05
High school	27.1	28.7	29.9	27.4	0.3	0.21 (0.20–0.22)	0.74
Apprenticeship or trade certificate	5.6	5.6	6.6	5.3	-0.3	0.02 (0.01–0.03)	0.97
College certificate or diploma, University certificate	24.2	23.9	25.9	23.1	-1.2	-0.14 (-0.15 –-0.13)	0.83
University undergraduate degree	17.6	16.3	13.5	16.7	-0.9	-0.40 (-0.41 –-0.39)	0.53
University graduate degree or Speciality (i.e., MD, JD)	9.3	8.2	5.8	8.9	-0.4	-0.30 (-0.31 –-0.29)	0.64
**Household Income, CAD**							
<$30,000	8.7	10.8	12.1	16.6	7.9	0.97 (0.97–0.97)	< 0.05
$30,000 –$49,999	12.4	13.4	13.9	17.1	4.6	0.93 (0.93–0.94)	0.05
$50,000 –$99,999	30.7	32.3	33.3	34.7	4.0	1.00 (0.99–1.00)	< 0.05
$100,000 –$149,999	22.4	22.1	21.9	18.0	-4.4	-0.83 (-0.83 –-0.83)	0.12
$150,000 –$199,999	12.9	11.8	10.7	7.8	-5.1	-0.97 (-0.97 –-0.97)	< 0.05
≥$200,000	12.8	9.6	8.1	5.8	-7.1	-0.99 (-0.99 –-0.99)	< 0.05
**Labour Force Employment**							
Employed	87.9	87.6	88.4	86.3	-1.5	0.47 (0.47–0.47)	0.45
Unemployed	12.2	12.3	11.6	13.7	1.5	-0.58 (-0.59 –-0.57)	0.35
**Housing Security**							
Spending ≥30% of income on housing	19.9	23.2	22.2	26.5	6.6	0.89 (0.89–0.89)	< 0.05

**Note**: ED = emergency department, Q1 = quartile 1 (neighborhoods with the lowest avoidable ED visits), Q2 = quartile 2, Q3 = quartile 3, Q4 = quartile 4 (neighborhoods with the highest avoidable ED visits), CI = confidence interval, CAD = Canadian dollars.

## Discussion

Our study found significant correlations between neighborhoods with high rates of avoidable ED visits and lower socioeconomic status factors, including low education, low income, and housing insecurity. Geospatially, neighborhoods of residents with higher avoidable ED visits were unevenly distributed in proximity to the hospital, suggesting other underlying factors such as SES influence avoidable ED utilization aside from close distance.

Previous research has predominantly focused on non-urgent ED use or frequent ED users rather than specifically avoidable ED visits. It is well-supported that individuals with low SES are more likely to use ED services, but was unclear whether these visits were avoidable [6, 16, 17]. We found that neighborhoods closer to the ED had more avoidable ED visits, a result consistent with the literature on both ED visits generally and less urgent visits specifically [18–20]. We found males and middle-aged adults were more likely to use the ED for conditions that could have been managed in the community, results that are supported in past research [16]. Our findings on income inequality among Hamilton neighborhoods and its disproportionate influence on healthcare utilization are consistent with previous reports of Hamilton, Ontario [21, 22].

We observed a clear SES gradient influencing avoidable ED visits. Specifically, neighborhoods with lower education levels (i.e., not graduating high school) had significantly higher associations with avoidable ED visits. This may be due to lower health literacy and awareness of healthcare alternatives [23, 24]. Additionally, neighborhoods with higher avoidable ED visit rates had a higher proportion of low-income households, and fewer higher-income households. This finding is consistent with the logic that low-income individuals often face barriers to accessing primary care, such as an inflexible work schedule, lack of transportation, and childcare challenges [25]. Other notable factors correlated to neighborhoods with high avoidable ED use included living in close proximity to the ED, living alone, and certain housing types.

A possible explanation for these findings could be Ontario’s shortage of primary care physicians in neighborhoods with lower SES [21]. Individuals with lower SES already face greater challenges in navigating the healthcare system, which is worsened by limited primary care access. This, in turn, exacerbates their disproportionately higher disease burden compared to higher SES individuals, as they may have less knowledge or ability to access healthcare alternatives apart from the ED [2]. Consequently, individuals with lower SES could be turning to the ED for primary care more frequently, drawn by convenience of 24-hour healthcare services, no appointment requirements, and perceived superior care compared to primary care [22]. Additionally, EDs may also be perceived as more accommodating to diverse social, cultural, and economic backgrounds than primary care offices, underscoring the necessity for primary care settings to be inclusive care environments to reduce avoidable ED visits. However, literature also shows that EDs can be stigmatizing, causing patients to withhold important information or not receiving appropriate treatment [26, 27]. Thus, the belief that EDs are more accommodating than primary care may not be consistently accurate, and should be reduced in all healthcare settings [28]. Lastly, another contributing factor could be that, despite Ontario’s universal healthcare system, where physician reimbursement is not tied to patient SES, individuals with lower SES still experience reduced access to primary care compared to their higher SES counterparts, leading to greater reliance on the ED [29].

Health policy initiatives aimed at reducing avoidable ED visits should focus on neighborhoods with lower SES. Expanding access to all types of community care, including both physician and non-physician (i.e., nurse practitioner, community paramedics), could be critical to mitigating avoidable ED attendance. Regardless of the current state of primary care in lower SES neighborhoods, improving primary care access could reduce avoidable ED attendance by facilitating regular health check-ups and screenings, thereby helping to prevent the onset of illness or enable earlier detection of conditions [30, 31]. Moreover, primary care providers can guide patients in navigating the health services, including specialist referrals and community resources, further reducing avoidable ED visits [32]. Lastly, targeted primary health initiatives could address the specific health needs of low SES populations through non-medical programs.

Future research should examine the associations between lower SES and avoidable ED visits at the patient and population levels in a universal healthcare system. Such studies could provide deeper insights into the complex interplay between SES and avoidable ED visits, informing more effective interventions and policies to reduce healthcare disparities and improve access to primary care for all socioeconomic groups.

### Limitations

Our analysis could not include patient-level SES factors due to data unavailability. Our analysis of neighborhoods relied on data from the Statistics Canada Census of Population 2021, which may contain a small degree of inaccuracies due to false responses or omit those without a residence address (i.e., homeless individuals). Additionally, our analysis was based on records from a single academic hospital; incorporating data from multiple hospital sites could strengthen the robustness of the results.

## Conclusion

Neighborhoods with high rates of avoidable ED utilization were correlated with lower SES, characterised by lower education, lower income and housing instability. Further analyses are required to inform targeted interventions in these disadvantaged areas, with the goal of reducing avoidable ED visits and fostering more equitable healthcare access and utilization.

## Supporting information

S1 Table STROBE statement. Reporting guideline for retrospective cohort studies.
(DOC)
